# Alexithymia in adolescents treated for psychiatric disorders: A study of the relationship with Alexithymia in parents and with the parental link

**DOI:** 10.1192/j.eurpsy.2023.1507

**Published:** 2023-07-19

**Authors:** D. Mnif, I. Hadjkacem, R. Sellami

**Affiliations:** 1 Child and adolescent Psychiatric department; 2 Child and adolescent psychiatric departement; 3 Adult psychiatric department, Hôpital Hedi chaker, Sfax, Tunisia

## Abstract

**Introduction:**

Alexithymia is defined as a disorder involving difficulties in identifying, describing feelings and an outward-oriented way of thinking with restricted imaginary processes. Our objectives were to study the link between parents’ alexithymia and that of adolescents treated for psychiatric disorders on the one hand, and to study the relationship between alexithymia in adolescents and the parental link perceived by them, on the other hand.

**Objectives:**

To study the relationship between the alexithymia of parents and that of adolescents followed for psychiatric disorders on the one hand, and to study the relationship between alexithymia in adolescents and the parental bond perceived by them on the other go.

**Methods:**

A descriptive and analytical cross-sectional study was carried out at the pediatric psychiatry consultation at the Hédi Chaker University Hospital in Sfax during a period of one year from January 2019 to December 2019. We recruited 116 people: 53 adolescents, 44 mothers and 19 fathers. . We used an information sheet, the TAS-20 score (Toronto Alexithymia Scale) and the PBI (Parental Bonding Instrument)

**Results:**

Two-thirds of the patients were alexithymic. A climate of domestic violence increased the adolescent’s emotional difficulties. Half of the mothers and one-third of the fathers were alexithymic. The total alexithymia score (TAS-20) in adolescents was positively correlated with the total alexithymia score (TAS-20) in the parents (p=0.03 for the mother and p = 0.02 for the father Adolescent difficulty in identifying emotions (DIE) was associated with difficulty describing emotions (DDE) in both parents (p=0.01 for mother and p<10-3 for father). The teenager had difficulty describing his own emotions when his mother was having the same difficulty. Outward thinking in adolescents were associated with more alexithymic traits in the mother. Maternal care scores were significantly and negatively correlated with adolescent TAS-20, DIE, and DDE scores (p=0.01, p=0.01, and p=0.04). Maternal protection score was positively and significantly correlated with TAS-20, DIE, and DDE scores (p=0.01, p=0.01 and p=0.02). The father’s protection score was positively and significantly correlated with TAS-20, DIE and DDE scores (p<10-3, p=0.04 and p=0.01). The overprotective attitude of parents perceived by the adolescent has therefore been associated with more alexithymia in adolescents.

**Conclusions:**

Particular attention must therefore be paid to the emotional difficulties of adolescents followed in child psychiatry, as well as those present with their parents. These difficulties can create interpersonal difficulties and affect the parental bond perceived by the child.

**Disclosure of Interest:**

None Declared

